# Green-Synthesized Silver Nanoparticles from *Zingiber officinale*: Physicochemical Characterization, Antibacterial Activity, and TMPRSS2-Modulating Potential

**DOI:** 10.3390/nano16140836

**Published:** 2026-07-08

**Authors:** Ozlem Tavukcuoglu, Fatih Ciftci, Nilüfer Evcimen Duygulu, Duygu Misirli, Mahfuz Elmastaş, Ahmet Akif Kızılkurtlu

**Affiliations:** 1 Department of Biochemistry, Faculty of Hamidiye Pharmacy, University of Health Sciences, Istanbul 34668, Turkey; ozlemoztolan@gmail.com (O.T.); duygu.misirli@sbu.edu.tr (D.M.); mahfuz.elmastas@sbu.edu.tr (M.E.); 2 Faculty of Engineering, Department of Biomedical Engineering, Fatih Sultan Mehmet Vakıf University, Istanbul 34445, Turkey; fciftci@fsm.edu.tr; 3 Biomedical Electronic Design Application and Research Center (BETAM), Fatih Sultan Mehmet Vakıf University, Istanbul 34445, Turkey; 4 BioriginAI Research Group, Department of Biomedical Engineering, Fatih Sultan Mehmet Vakıf University, Istanbul 34445, Turkey; 5 Faculty of Chemical and Metallurgical Engineering, Department of Metallurgical and Material Engineering, Yildiz Technical University, Istanbul 34469, Turkey; nevci@yildiz.edu.tr; 6 Faculty of Engineering and Natural Sciences, Department of Biomedical Engineering, Atlas University, Istanbul 34408, Turkey

**Keywords:** *Zingiber officinale*, green synthesis, TMPRSS2 inhibition, silver nanoparticles

## Abstract

In this study, green-synthesized silver nanoparticles derived from *Zingiber officinale* (G-AgNPs) were investigated as potential modulators of transmembrane serine protease 2 (TMPRSS2), a host-associated protease involved in viral entry mechanisms. Before nanoparticle synthesis, the phytochemical composition of ginger extract was analyzed using high-performance liquid chromatography (HPLC) with photodiode array detection. Silver nanoparticles were synthesized using aqueous ginger extract as a reducing and stabilizing agent. The nanoparticles were characterized by ultraviolet–visible spectroscopy (UV–Vis.), Fourier transform infrared spectroscopy (FT-IR), dynamic light scattering (DLS), zeta potential analysis, X-ray diffraction (XRD), and transmission electron microscopy (TEM). The synthesized silver nanoparticles exhibited a face-centered cubic (*fcc*) crystalline structure, nanoscale particle size distribution, and moderate colloidal stability. Transmission electron microscopy revealed predominantly quasi-spherical nanoparticles with an average diameter of 10.61 ± 1.31 nm, while X-ray diffraction indicated an average crystallite size of 15.28 ± 5.48 nm. Biological evaluation demonstrated robust, broad-spectrum antibacterial activity against Gram-negative *Escherichia coli* and Gram-positive *Staphylococcus aureus*, with distinct susceptibility profiles. Minimum Inhibitory Concentration (MIC) values were 3.125 µg/mL and 12.5 µg/mL, and Minimum Bactericidal Concentration (MBC) values were 6.25 µg/mL and 25.0 µg/mL, respectively. Cell culture assays confirmed high cytocompatibility with L929 fibroblasts at all tested concentrations. In a fluorometric enzyme assay, the silver nanoparticles inhibited TMPRSS2 activity in a concentration-dependent manner, achieving 51.24% inhibition at 100 µg/mL and an estimated IC50 of 40.06 µg/mL. Although the inhibitory activity was lower than that of Camostat, the findings suggest that ginger-mediated silver nanoparticles represent promising plant-based nano-bioactive systems for further investigation of TMPRSS2 modulation.

## 1. Introduction

Plant-derived bioactive compounds have attracted considerable attention in biomedical research due to their diverse biological activities and expanding applications in nanotechnology. Green synthesis methods using plant extracts offer an environmentally sustainable and cost-effective alternative to conventional physical and chemical nanoparticle production techniques, which often require toxic reagents, high energy inputs, and harsh reaction conditions [1,2]. In plant-mediated synthesis, phytochemicals serve as reducing and stabilizing agents, as well as bioactive surface-functionalizing molecules that enhance the colloidal stability and biological properties of nanoparticles [3,4]. These compounds possess unique physicochemical properties and exhibit a broad spectrum of biological activities, including antimicrobial, antioxidant, anti-inflammatory, and antiviral effects [5,6].

The biological performance of green-synthesized silver nanoparticles (AgNPs) is largely determined by the phytochemical composition of the plant extract employed during synthesis, which governs not only nanoparticle formation and stability but also their surface chemistry and biological functionality. Owing to this unique phytochemical functionalization, AgNPs have emerged as one of the most versatile nanomaterials for biomedical applications, exhibiting intrinsic antimicrobial, antioxidant, anti-inflammatory, antiviral, and enzyme-modulating properties [7]. Their multifunctional nature enables integration of structural stability and biological activity without extensive post-synthesis surface modification. In contrast, other inorganic nanomaterials, such as silica nanoparticles (SiO_2_ NPs), are recognized for their chemical stability, tunable pore structure, high surface area, and suitability as drug-delivery platforms [8]. Similarly, green-synthesized metallic and metal oxide nanoparticles, including gold (Au) [9], copper (Cu) [10], zinc oxide (ZnO) [11,12], and titanium dioxide (TiO_2_) [13], have demonstrated promising performance in photothermal therapy, tissue engineering, bioimaging, and targeted drug delivery. However, these nanomaterials generally exhibit application-specific functionalities and often require additional surface engineering or incorporation of bioactive molecules to achieve enhanced therapeutic efficacy. In contrast, green-synthesized AgNPs combine the biological activity of metallic silver with phytochemical capping agents from medicinal plants, providing broad-spectrum antimicrobial activity and the potential to modulate biologically relevant molecular targets [14].

Ginger (*Zingiber officinale*) is among the most extensively studied medicinal plants due to its rich phytochemical profile, particularly gingerols, shogaols, paradols, and other phenolic constituents, which exhibit antioxidant, anti-inflammatory, immunomodulatory, and antiviral activities. Nevertheless, the therapeutic application of ginger-derived compounds is frequently constrained by poor aqueous solubility, susceptibility to oxidative degradation, and low bioavailability under physiological conditions [15,16]. As a result, nanoparticle-based formulations have attracted increasing interest as an effective strategy to enhance the stability, bioavailability, and functional activity of ginger-derived bioactive compounds [17,18].

Recent studies have demonstrated the successful green synthesis of silver nanoparticles using *Zingiber officinale* extracts and have highlighted their biomedical potential. Ongtanasup et al. [19] reported that ginger-mediated AgNPs exhibit significant antioxidant activity, anti-lipoxygenase (LOX) potential, biocompatibility, and favorable molecular docking interactions, supporting their applicability as anti-inflammatory nanomaterials. Similarly, Ramzan et al. [20] synthesized ginger-derived AgNPs and demonstrated their antibacterial efficacy against multidrug-resistant bacterial pathogens following comprehensive physicochemical characterization. While these studies highlight the biomedical potential of ginger-mediated AgNPs, most investigations have concentrated on antimicrobial, antioxidant, anti-inflammatory, and cytocompatibility assessments. The interaction of ginger-mediated AgNPs with host-associated molecular targets involved in viral infection remains unexplored, representing a notable gap in the current literature.

Transmembrane serine protease 2 (TMPRSS2) is a host-associated protease essential for the activation and cellular entry of several respiratory viruses, including coronaviruses and influenza viruses [21,22]. Unlike conventional antiviral strategies that target viral proteins, modulation of host proteins such as TMPRSS2 has emerged as a promising therapeutic approach, potentially reducing the likelihood of viral resistance and broadening antiviral applicability. Recent evidence suggests that certain plant-mediated nanoparticles possess enzyme-modulating properties and can disrupt host-associated biological pathways [23]. Additionally, green-synthesized AgNPs from medicinal plants such as Sambucus nigra and Arbutus unedo have demonstrated preliminary inhibitory activity against TMPRSS2 [24,25]. Despite extensive research on the antimicrobial and biomedical properties of ginger-mediated AgNPs, their ability to inhibit TMPRSS2 has not been investigated. Expanding the range of phytochemical-based TMPRSS2-modulating nanomaterials may facilitate the development of future host-targeted nano-enabled biomedical strategies [26].

Therefore, this study aimed to synthesize silver nanoparticles using *Zingiber officinale* extract (G-AgNPs) via an environmentally friendly green synthesis approach and to comprehensively characterize their physicochemical properties. Before nanoparticle synthesis, the phenolic profile of the ginger extract was analyzed by HPLC-PDA to establish the phytochemical basis for nanoparticle formation. The synthesized nanoparticles were then characterized using complementary physicochemical techniques and evaluated for antibacterial activity, cytocompatibility, and inhibitory activity against TMPRSS2. In contrast to previous studies, which primarily focused on the antimicrobial, antioxidant, or anti-inflammatory properties of ginger-mediated AgNPs, the present work specifically examines their ability to modulate TMPRSS2, a host-associated enzyme critical for viral entry. To the best of our knowledge, this is the first study to report the TMPRSS2-inhibitory activity of *Zingiber officinale*-mediated silver nanoparticles, thereby expanding the biomedical applications of ginger-derived AgNPs from conventional antimicrobial nanomaterials to multifunctional, host-targeted, nano-bioactive systems.

## 2. Materials and Methods

### 2.1. Plant Materials and Chemicals

Fresh ginger rhizomes (*Zingiber officinale*) used for the green synthesis of silver nanoparticles were obtained from a local organic market in Istanbul, Turkey. The botanical identity of the plant material was verified based on its characteristic morphological features before use. All plant materials were thoroughly washed with distilled water to remove surface impurities, then sliced into small pieces and processed immediately after purchase to preserve their phytochemical integrity.

Silver nitrate (AgNO_3_; molecular weight: 169.87 g/mol; purity: 99%) and ethanol (≥99.5% purity) were purchased from Sigma-Aldrich (Darmstadt, Germany) and used as received without further purification. Ultrapure water (resistivity: 18.2 MΩ·cm) was generated using a Millipore Direct-Q^®^ 3 water purification system (Merck Millipore, Darmstadt, Germany) for all experimental steps.

The murine fibroblast cell line L929, employed for cytotoxicity assays, was obtained from the American Type Culture Collection (ATCC, Manassas, VA, USA). Cell culture media and supplements, including Dulbecco’s Modified Eagle’s Medium/Nutrient Mixture F-12 (DMEM/F12), fetal bovine serum (FBS), trypsin-EDTA (0.25%), and Trypan Blue, were procured from Gibco (Thermo Fisher Scientific, Grand Island, NY, USA).

Additional reagents such as penicillin-streptomycin antibiotic solution, phosphate-buffered saline (PBS) tablets, XTT [2,3-bis-(2-methoxy-4-nitro-5-sulfenyl)-2H-tetrazolium-5-carboxanilide], and phenazine methosulfate (PMS) were supplied by Santa Cruz Biotechnology (Dallas, TX, USA). The TMPRSS2 Fluorogenic Assay Kit, utilized to evaluate enzyme inhibition, was purchased from BPS Bioscience (San Diego, CA, USA).

### 2.2. Preparation of Zingiber officinale Extract

Fresh *Zingiber officinale* rhizomes were thoroughly rinsed with tap water to eliminate visible soil and debris, then rinsed a final time with ultrapure water to remove residual impurities. The cleaned rhizomes were gently blotted dry and air-dried at ambient temperature (~24 °C) to remove surface moisture. Once dried, the plant material was finely chopped and homogenized using a mechanical grinder. A total of 10 g of ground ginger (10%, *w*/*v*) was placed into a 250 mL borosilicate beaker containing 100 mL of ultrapure water. The suspension was magnetically stirred at 80 °C for 20 min to facilitate extraction of thermally stable phytochemicals. After heating, the mixture was allowed to cool and then stirred at room temperature for 60 min to promote further diffusion of bioactive constituents.

The extract was initially filtered through a fine-mesh strainer to remove any coarse plant material. To eliminate micron-scale particulate matter, the filtrate was centrifuged at 4000 rpm for 10 min. The clarified supernatant was then filtered through Whatman No. 1 filter paper, transferred to sterile amber vials, and stored at 4 °C. The final pH of the ginger extract was 5.3, consistent with the mildly acidic nature of phenolic-rich plant matrices.

### 2.3. Synthesis of Silver Nanoparticles Using Ginger (Zingiber officinale) Extract (G-AgNPs)

G-AgNPs were synthesized using a green synthesis approach with an aqueous extract of *Zingiber officinale*. Specifically, 3 mL of ginger extract (10%, *w*/*v*) was added dropwise to 7 mL of freshly prepared 1 mM AgNO_3_ solution. Before mixing, the pH of the AgNO_3_ solution was adjusted to 8.0 using a dilute NaOH solution.

The reaction mixture was magnetically stirred at 700 rpm and maintained at 45 °C for 2 h. After synthesis, the nanoparticles were collected by centrifugation at 12,000 rpm for 20 min at 4 °C and washed twice with ultrapure water to remove residual phytochemicals and unreacted precursors. The purified G-AgNPs were first frozen at −80 °C and then lyophilized at −50 °C for 24 h under vacuum using a HyperCOOL HC3055 freeze-dryer (Daejeon, Republic of Korea). The lyophilized nanoparticles were stored at 4 °C in amber vials until further characterization and biological assays.

### 2.4. Characterization

The physicochemical and structural characteristics of the synthesized G-AgNPs were comprehensively evaluated using a combination of spectroscopic, microscopic, chromatographic, and scattering-based techniques. UV–Vis spectroscopy (UV-1900, Shimadzu, Kyoto, Japan) was employed to monitor the formation and optical behavior of silver nanoparticles. Spectra were recorded over a wavelength range of 200–800 nm at specific intervals during synthesis to monitor the development of the surface plasmon resonance (SPR) band. The hydrodynamic diameter of the nanoparticles was determined using a Zetasizer Nano ZS (Malvern Instruments Ltd., Malvern, UK), following dilution in ultrapure water and brief sonication to minimize agglomeration.

FT-IR spectroscopy was conducted using a Thermo Scientific Nicolet™ Continuµm™ Infrared Microscope (Thermo Fisher Scientific Inc., Waltham, MA, USA). Spectra were recorded over the range of 4000–400 cm^−1^ to identify functional groups present in the ginger extract and to assess their possible involvement in the reduction and stabilization of Ag^+^ ions. TEM was performed to examine the morphology and size distribution of G-AgNPs. A drop of diluted nanoparticle suspension was placed onto carbon-coated copper grids (CF200-Cu, 200-mesh, Electron Microscopy Sciences, Hatfield, PA, USA) and dried at room temperature before imaging. Images were acquired with a Gatan Model 833 Orius SC200D CCD camera. Nanoparticle sizes were measured using ImageJ software (version 1.53t, National Institutes of Health, Bethesda, MD, USA), with 100 measurements recorded per sample.

XRD analysis was performed using a Bruker D8 Advance diffractometer (Bruker Corporation, Billerica, MA, USA) with Cu Kα radiation (λ = 1.5406 Å), operated at 40 kV and 30 mA. Data were collected over a 2θ range of 10° to 90° with a step size of 0.02°. The average crystallite size of the nanoparticles was calculated using the Debye–Scherrer equation applied to the full width at half maximum (FWHM) of major diffraction peaks, consistent with the polycrystalline nature of the nanoparticles [27].

Additionally, HPLC analysis was conducted to determine the phenolic composition of the ginger extract before nanoparticle synthesis. The analysis was performed on a Shimadzu Nexera-i LC-2040C 3D Plus (Shimadzu Corporation, Kyoto, Japan) system equipped with a photodiode array (PDA) detector set at 254 nm. Compound-specific λmax values were additionally confirmed against authentic standards (Table S1, Supplementary Information). Chromatographic separation was performed on a phenyl-hexyl analytical column (4.6 × 150 mm, 3 μm, GL Sciences InterSustain, Tokyo, Japan) using a binary solvent gradient.

The mobile phase consisted of Solvent A (0.1% formic acid in water) and Solvent B (acetonitrile), applied under a detailed binary solvent gradient: 95:5 (A:B) at 0 min, 90.5:9.5 at 7 min, 83:17 at 20 min, 40:60 at 35 min, and 100:0 at 40 min. The flow rate was 1.0 mL/min, the injection volume was 10 µL, and the column temperature was maintained at 30 °C.

For compound identification and quantification, 15 authentic phenolic standards were analyzed under identical conditions, and each peak was matched by retention time and UV–Vis absorption maxima. Calibration curves with R^2^ > 0.99 were used for quantification, and LOD/LOQ values are provided in Supplementary Table S1. The representative HPLC chromatogram is shown in Supplementary Figure S1. The method was validated previously for accuracy and reproducibility [28,29].

### 2.5. Antibacterial Analysis

The antibacterial potential of GE and G-AgNPs was assessed using the agar disk diffusion method following established protocols [30,31]. *Escherichia coli* (*E. coli*, ATCC 25922) and *Staphylococcus aureus* (*S. aureus*, ATCC 25923) were selected as the representative Gram-negative and Gram-positive model strains, respectively. The bacterial cultures were maintained on Mueller–Hinton Agar (MHA; Oxoid) and subcultured overnight in Mueller–Hinton Broth (MHB; Oxoid) at 37 °C prior to each assay. GE and G-AgNPs were prepared in sterile distilled water or appropriate analytical solvents (DMSO ≤ 1%, when necessary). Final test concentrations were adjusted to 25, 50, and 100 mg/mL for the extracts, and to 10, 25, and 50 µg/mL (calculated based on the dry weight of AgNPs) for the nanoparticle formulations. All test solutions were filtered through 0.22 μm membrane filters and exposed to UV light for 2 h to ensure sterility.

Sterile blank paper disks (6 mm diameter; Oxoid) were impregnated with 20 µL of each sample and dried under aseptic conditions. The disks were then equidistantly placed onto the surface of MHA plates previously inoculated with bacterial suspensions adjusted to a 0.5 McFarland standard (~1.5 × 10^8^ CFU/mL). Disks were positioned 22 mm apart and 14 mm from the Petri dish margin to prevent overlapping zones of inhibition. Amoxicillin (10 µg/disk) served as the positive control, while disks impregnated with the respective solvents only (sterile water or 1% DMSO) were utilized as negative controls. The plates were incubated at 37 °C for 18–24 h. Post-incubation, the diameters of the inhibition zones were measured in millimeters using a digital caliper. The experimental outputs were interpreted in accordance with the Clinical and Laboratory Standards Institute (CLSI) guidelines (M02-A12). All assays were performed in triplicate, and the data were expressed as mean ± standard deviation.

#### Determination of MIC and MBC

The Minimum Inhibitory Concentration (MIC) and Minimum Bactericidal Concentration (MBC) of GE and G-AgNPs were determined using the broth microdilution method in 96-well microplates, in accordance with the Clinical and Laboratory Standards Institute (CLSI) guidelines. Two-fold serial dilutions of the samples were prepared in Mueller–Hinton Broth, yielding concentration ranges of 3.125–200 mg/mL for GE and 1.25–80 µg/mL for G-AgNPs. Each well was inoculated with a standardized bacterial suspension to achieve a final inoculum density of 5 × 10^5^ CFU/mL. The microplates were incubated under aerobic conditions at 37 °C for 24 h. The MIC was defined as the lowest concentration of the respective agent required to completely inhibit visible bacterial growth. To determine the MBC, 10 µL aliquots from all wells showing no visible growth were subcultured onto fresh Mueller–Hinton Agar plates and incubated at 37 °C for an additional 24 h. The MBC was recorded as the lowest concentration that reduced the initial bacterial inoculum by ≥99.9%. All quantitative assays were executed in triplicate.

### 2.6. In Vitro Cytotoxicity Analyses

In vitro cytotoxicity evaluations were performed using L929 murine fibroblast cells in accordance with ISO 10993-5 standards. The aim was to assess the potential cytotoxic effects of the GE and G-AgNPs.

L929 cells were cultured in Dulbecco’s Modified Eagle’s Medium/Nutrient Mixture F-12 (DMEM/F12), supplemented with 10% fetal bovine serum (FBS), 10,000 U/mL penicillin, and 10 mg/mL of streptomycin, along with an additional 0.5% penicillin–streptomycin solution. The cells were maintained at 37 °C in a humidified incubator with a 5% CO_2_ atmosphere to ensure optimal growth conditions.

Upon reaching confluence, the cells were detached using trypsin–EDTA solution and centrifuged at 1000 rpm for 5 min. The supernatant was discarded, and the viable cells in the pellet were counted using a hemocytometer to adjust the appropriate seeding density for the cytotoxicity tests. The direct and indirect XTT-based cytotoxicity assays were conducted as described previously [32].

#### 2.6.1. Direct Contact Method (XTT Assay)

For the direct contact assay, L929 murine fibroblasts were seeded into 48-well flat-bottom microplates at a density of 3 × 10^4^ cells per well in 500 µL of complete growth medium and incubated at 37 °C with 5% CO_2_ for 24 h to ensure baseline cellular attachment. Concurrently, test samples were prepared by dispersing the lyophilized G-AgNPs or raw extracts in sterile distilled water, with brief sonication (using analytical-grade DMSO at ≤1% when necessary to dissolve crude extracts). The stock suspensions were then introduced into the respective wells to achieve final operational concentrations of 25, 50, and 100 µg/mL (calculated based on AgNP dry weight or crude extract equivalent).

Following the exposure period, the culture medium was completely aspirated from each well and replaced with 500 µL of freshly prepared XTT working solution containing 0.5 mg/mL of 2,3-bis-(2-methoxy-4-nitro-5-sulfophenyl)-2H-tetrazolium-5-carboxanilide (XTT) and 7.5 mg/mL of phenazine methosulfate (PMS) electron coupling reagent. The microplates were incubated for an additional 4 h at 37 °C to allow for the mitochondrial bioconversion of XTT into water-soluble formazan crystals. The optical density (OD) of each well was subsequently measured at 450 nm using a microplate reader.

Cell culture medium without any test materials or vehicle artifacts served as the negative control (0 µg/mL), establishing the 100% viability reference benchmark. In accordance with comparative screening frameworks for biogenic nanomaterials, an independent chemical lytic agent was omitted as a positive control because the technical objective was centered on mapping the relative, non-destructive biocompatibility index of G-AgNPs directly against an optimal, uninhibited metabolic baseline. Quantitative cell viability percentages were calculated from the recorded absorbance values, relative to the untreated control group, to precisely determine cytotoxicity under direct-contact exposure conditions.

#### 2.6.2. Indirect Contact Method (XTT Assay)

For the indirect assay, varying volumes (25 µL, 50 µL, 75 µL, and 100 µL) of G-AgNPs and GE samples were incubated in 500 µL of complete cell culture medium for 24 h at 37 °C to allow leachable components to diffuse. After incubation, the conditioned media were applied to L929 cells previously seeded into 96-well plates (1 × 10^4^ cells/well), and the cells were incubated for an additional 24 h.

Following this incubation period, 100 µL of XTT reagent was added to each well, and plates were incubated for 4 h. Absorbance was measured at 450 nm, and cell viability percentages were calculated relative to the untreated control group to assess potential cytotoxicity induced by leachates from the tested samples [33,34]. The conditioned media corresponded to equivalent G-AgNPs or extract concentrations of 25, 50, 75, and 100 µg/mL, with AgNP formulations dispersed in sterile distilled water by sonication and extracts dissolved in sterile distilled water (DMSO ≤ 1% when necessary).

### 2.7. Fluorometric Evaluation of TMPRSS2 Enzyme Inhibition

The inhibitory activity of the GE and G-AgNPs against the TMPRSS2 enzyme was evaluated using a fluorometric TMPRSS2 assay kit (TMPRSS2 Fluorogenic Assay Kit, BPS Bioscience, San Diego, CA, USA, Cat. No: 78083) according to the manufacturer’s protocol.

Assays were conducted in a 96-well microplate format. Each well was filled with 50 μL of assay buffer and 10 μL of TMPRSS2 enzyme solution. Experimental wells received either G-AgNPs or camostat (a reference inhibitor) at various concentrations to assess their inhibitory potential. Negative control wells contain inhibitor-free buffer. For this assay, G-AgNPs were re-suspended in 5% DMSO to ensure compatibility with the kit buffer, while extracts were dissolved in ultrapure water.

The enzymatic reaction was initiated by adding 40 μL of the fluorogenic substrate solution, resulting in a final reaction volume of 100 μL per well. The plate was incubated at 37 °C, and fluorescence readings were recorded every 2 min for 30 min. The excitation and emission wavelengths were set at 350 nm and 450 nm, respectively. All experimental groups, including controls, were evaluated under identical assay conditions to minimize potential analytical variability associated with fluorescence-based measurements.

The percentage inhibition of TMPRSS2 enzymatic activity was calculated by comparing the fluorescence intensities of the treated samples to those of the control group. This quantitative approach enabled real-time monitoring of enzyme inhibition and provided a reproducible framework for evaluating the potential of G-AgNPs as TMPRSS2 inhibitors. G-AgNPs were tested at concentrations of 25, 50, and 100 µg/mL, calculated on a dry-weight basis. In contrast, the corresponding plant extracts were tested at equivalent concentrations (25, 50, and 100 µg/mL of crude extract). The fluorogenic peptide-based assay format has been widely used in studies of TMPRSS2 inhibitors [35].

### 2.8. Statistical Analysis

All statistical analyses were performed using GraphPad Prism software (version 8; GraphPad Software Inc., San Diego, CA, USA). Quantitative results are expressed as mean ± standard deviation (SD) from at least three independent experiments (*n* = 3).

For antibacterial and cytotoxicity assays, group comparisons were conducted using one-way analysis of variance (ANOVA), followed by Tukey’s multiple comparisons test to assess significant differences between treatments and control. Statistical significance levels were set at * *p* < 0.05, ** *p* < 0.01, and *** *p* < 0.001.

For TMPRSS2 enzyme inhibition assays, two-way ANOVA was applied to assess treatment and concentration effects, followed by a Bonferroni post hoc test for pairwise comparisons. In addition, a four-parameter logistic regression model was used to fit the dose–response curve of G-AgNPs. Comparisons against the standard inhibitor, camostat (50 nM), were indicated with hash symbols (# *p* < 0.05, ## *p* < 0.01, ### *p* < 0.001), as shown in the figure legends.

All graphical outputs were generated using OriginPro software (version 9; OriginLab Corporation, Northampton, MA, USA).

## 3. Results and Discussion

The synthesized G-AgNPs were comprehensively characterized to assess their phytochemical composition, optical properties, surface chemistry, morphology, crystallinity, particle-size distribution, and colloidal stability. HPLC was performed to characterize the phenolic profile of the ginger extract before nanoparticle synthesis. The formation and physicochemical properties of G-AgNPs were subsequently investigated using UV–Vis spectroscopy, FT-IR, TEM, HRTEM, DLS, zeta potential analysis, and XRD. Finally, the biological performance of G-AgNPs was assessed through antibacterial activity, cytocompatibility, and TMPRSS2 inhibition assays.

### 3.1. Characterization of Ge and G-AgNPs

**Figure 1:**
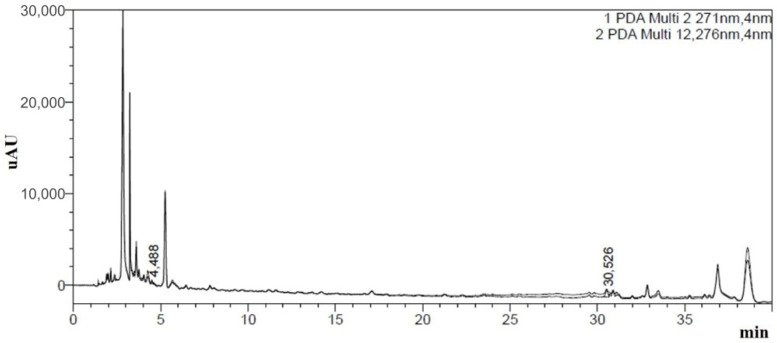
Representative HPLC–PDA chromatogram of GE, showing the separation profile of the detected phenolic compounds.

Figure 1 presents a representative HPLC-PDA chromatogram of the GE, showing distinct phenolic peaks. Based on chromatographic analysis, two phenolic compounds, gallic acid (12.2 μg/g extract) and cinnamic acid (15.6 μg/g extract), were identified and quantified, as summarized in Table 1.

The phytochemical profile of *Zingiber officinale* varies considerably depending on the cultivar, geographical origin, extraction conditions, and, especially, the extraction solvent. Due to differences in solvent polarity, aqueous extraction may not efficiently recover all phenolic constituents commonly reported in ethanolic or hydroalcoholic ginger extracts. Consequently, several phenolic compounds may be absent or remain below the limits of detection under the extraction conditions employed in the present study [36,37]. Furthermore, the principal bioactive constituents of ginger, gingerols and shogaols, were not included among the analytical standards used in the present HPLC method.

Gallic acid and cinnamic acid are well recognized for their antioxidant [38,39], anti-inflammatory [40], and antimicrobial [39,41] activities. In addition, these phenolic compounds have been reported to participate in the bioreduction of Ag^+^ ions and the stabilization of silver nanoparticles. Previous studies have demonstrated that even low concentrations of gallic acid and cinnamic acid can serve as effective electron donors during green nanoparticle synthesis [42,43,44,45].

However, plant-mediated nanoparticle synthesis is generally governed by the collective action of multiple phytochemicals rather than a single constituent. Therefore, the reduction of Ag^+^ ions and stabilization of G-AgNPs were likely achieved through the synergistic action of the detected phenolic compounds and other unquantified phytochemicals naturally present in the aqueous ginger extract. Collectively, these findings support the aqueous ginger extract as a source of phytochemicals that contributed to the reduction of Ag^+^ ions and stabilization of the synthesized G-AgNPs.

**Figure 2:**
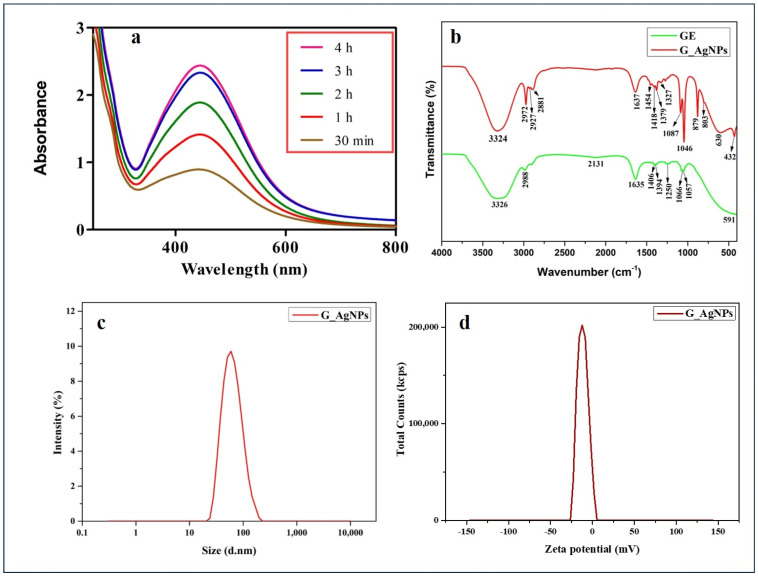
Characterization of G-AgNPs. (**a**) UV–Vis spectra recorded at different time intervals during nanoparticle synthesis; (**b**) FT-IR spectra of GE and G-AgNPs; (**c**) particle size distribution of G-AgNPs determined by DLS; and (**d**) zeta potential distribution of G-AgNPs.

UV–Vis spectroscopy analysis provided valuable information about the formation and optical properties of green-synthesized G-AgNPs, as shown in Figure 2a. Absorbance spectra were recorded at multiple time intervals (30 min, 1 h, 2 h, 3 h, and 4 h) throughout the synthesis process. A significant increase in intensity was observed over time, accompanied by a well-defined surface plasmon resonance (SPR) band centered at 449 nm, indicating the successful formation of silver nanoparticles. The gradual increase in the SPR peak reflects the accumulation of nanoparticles and confirms the reduction of Ag^+^ ions by the phytochemicals present in the ginger extract. This is consistent with the SPR phenomenon, where conduction electrons on the nanoparticle surface resonate with the incident light, and the absorption maximum is related to the nanoparticle size, shape, and concentration [46,47].

Figure 2b illustrates the FT-IR spectra of the GE and the synthesized G-AgNPs. The detailed FT-IR band assignments are summarized in Supplementary Table S2. In the GE spectrum, the major absorption bands correspond to hydroxyl, aliphatic C–H, carbonyl, C–N, and C–O-containing functional groups commonly associated with phenolic compounds and other plant-derived biomolecules.

Compared with the GE spectrum, the FT-IR spectrum of G-AgNPs exhibited several shifts together with newly appeared bands, suggesting the participation of phytochemical functional groups during nanoparticle formation. The slight shift in the broad O–H stretching band from 3326 to 3324 cm^−1^ indicates changes in the hydrogen-bonding environment, supporting the involvement of hydroxyl-containing phytochemicals during AgNP synthesis. Gallic acid, owing to the presence of three hydroxyl groups and one carboxyl group, is considered an efficient electron donor that may facilitate Ag^+^ reduction [43,45]. Following nanoparticle formation, these hydroxyl and carboxyl functionalities are likely adsorbed onto the nanoparticle surface, contributing to steric and electrostatic stabilization [48]. Similarly, cinnamic acid, containing a carboxyl group conjugated to an aromatic ring, may also participate in surface adsorption and enhance colloidal stability. In addition to these identified phenolics, other oxygen-containing phytochemicals naturally present in the aqueous ginger extract may also participate in Ag^+^ coordination, surface adsorption, and nanoparticle stabilization.

Notably, the bands at 1637, 1454, 1418, 1379, and 1327 cm^−1^ exhibited changes in intensity and position relative to the GE spectrum, indicating alterations in the local chemical environment of carbonyl- and hydroxyl-containing biomolecules following nanoparticle formation. Likewise, the newly appeared bands at 1087, 1046, 879, 803, 630, and 432 cm^−1^ further support the adsorption of plant-derived biomolecules onto the nanoparticle surface. Nevertheless, plant-mediated nanoparticle synthesis is governed by the collective action of multiple phytochemicals rather than a single constituent. Therefore, the reduction in Ag^+^ ions and stabilization of G-AgNPs were most likely achieved through the synergistic action of the detected phenolic compounds together with other unquantified phytochemicals present in the aqueous ginger extract. These FT-IR observations are consistent with previous reports on plant-mediated AgNP synthesis, in which multiple phytochemicals function synergistically as reducing and capping agents [36,49].

Figure 2c shows the DLS results for G-AgNPs, indicating a sharp, narrow, monomodal size distribution with a Z-average diameter of 80.06 ± 9.8 nm. This relatively narrow size range indicates a consistent particle population, which is essential for reproducibility and application-specific performance. The polydispersity index (PDI) was 0.3092, indicating moderate size uniformity and acceptable colloidal stability. Furthermore, the zeta potential was measured as −15.06 mV, reflecting moderate electrostatic stabilization of the colloidal system [50]. Although this value does not indicate a strong repulsion, it may be sufficient when combined with the steric stabilization provided by the phytochemicals in the ginger extract. These findings together confirm the successful synthesis of moderately stable, monodisperse silver nanoparticles through a green approach based on GE.

### 3.2. Structural Characterization of G-AgNPs

**Figure 3:**
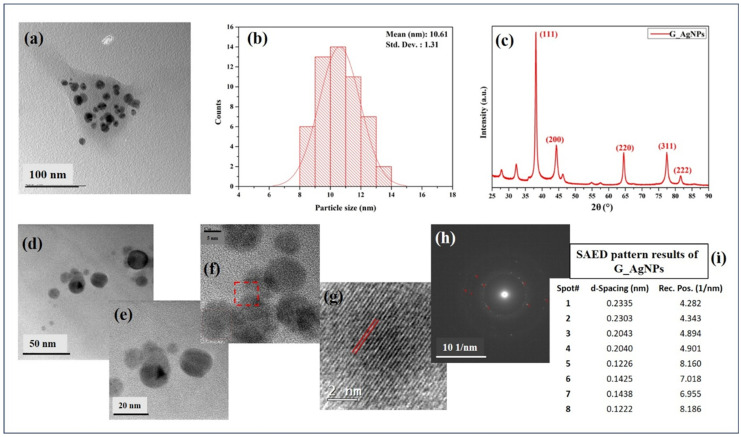
Structural characterization of G-AgNPs. (**a**) TEM image; (**b**) particle size distribution histogram obtained from TEM measurements (*n* = 100 particles); (**c**) XRD pattern; (**d**–**f**) HRTEM images recorded at different magnifications; (**g**) lattice fringes; (**h**) SAED pattern; and (**i**) corresponding SAED results.

TEM analysis provided detailed insights into the morphology and structure of the synthesized G-AgNPs. As shown in Figure 3a, the nanoparticles exhibited predominantly quasi-spherical shapes with good dispersion and minimal aggregation. The size distribution of the nanoparticles, determined from multiple TEM images and summarized in Figure 3b, ranged from approximately 6 to 14 nm, with an average diameter of 10.61 ± 1.31 nm. This narrow size distribution suggests the successful formation of a monodisperse nanoparticle population, which is essential for ensuring consistency in various biomedical and catalytic applications [51].

Higher-magnification TEM images (Figure 3d–f) confirm the uniformity in particle morphology. The high-resolution HR-TEM image in Figure 3g reveals resolved lattice fringes, which confirm the crystalline nature of the nanoparticles. The observed lattice spacing (*d* = 0.204 nm) corresponds to the (200) plane of silver, indicating a face-centered cubic (*fcc*) structure. These findings are further supported by the Selected Area Electron Diffraction (SAED) pattern shown in Figure 3h, which displays concentric diffraction rings characteristic of a polycrystalline structure. The measured d-spacing values, listed in Figure 3i, closely match standard silver diffraction data, further confirming the crystalline nature of the nanoparticles [52].

The structural characteristics of G-AgNPs were further confirmed by XRD analysis (Figure 3c). The XRD pattern showed five prominent diffraction peaks at 2θ = 38.12°, 44.31°, 64.46°, 77.41°, and 81.56°, corresponding to the (111), (200), (220), (311), and (222) planes, respectively. These reflections match the standard pattern for face-centered cubic silver (JCPDS No. 89-3722), indicating the successful synthesis of highly crystalline silver nanoparticles. The crystallite sizes calculated using the Scherrer equation for each diffraction peak are presented in Table 2 [53]. The average crystallite size was 15.28 nm, with the (111) plane exhibiting the largest individual size of 24.88 nm, indicating preferential growth along this facet.

These results are consistent with previous studies that report similar *fcc* crystalline structures and XRD peak positions in green-synthesized silver nanoparticles [54,55].

A comparison of the physicochemical information obtained from TEM, HRTEM, XRD, and DLS is provided in Supplementary Table S3. These characterization techniques provide complementary rather than contradictory information because each measures a different structural or physicochemical property of the nanoparticles. The discrepancy between the primary particle size determined by TEM (10.61 ± 1.31 nm) and the hydrodynamic diameter measured by DLS (80.06 ± 9.8 nm) is expected and arises from the different physical principles underlying these characterization techniques. TEM measures the metallic nanoparticle core in the dry state, whereas XRD determines the average crystallite size of the crystalline domains, and HRTEM confirms the crystalline lattice at the atomic scale. In contrast, DLS measures the hydrodynamic diameter of nanoparticles dispersed in solution. Consequently, the DLS measurement includes not only the metallic core but also the surrounding hydration shell, the phytochemical coating layer adsorbed onto the nanoparticle surface during green synthesis, and possible mild aggregation in colloidal suspension. Similar differences between TEM and DLS measurements have frequently been reported for plant-mediated AgNPs because DLS reflects the behavior of nanoparticles in their native colloidal environment rather than the size of individual metallic cores [2,56].

### 3.3. Antibacterial Analysis of G-AgNPs

**Figure 4:**
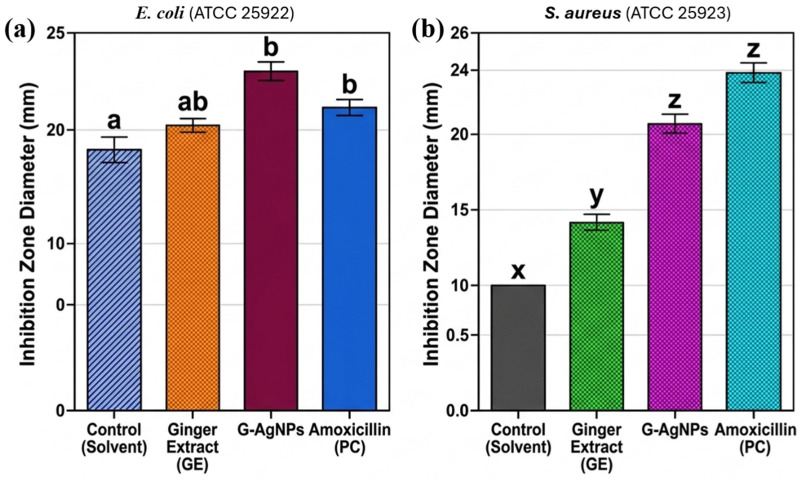
Quantitative evaluation of inhibition zone diameters (mm) for the solvent control, GE, biosynthesized G-AgNPs, and the positive control (amoxicillin) against (**a**) *Escherichia coli* (ATCC 25922) and (**b**) *Staphylococcus aureus* (ATCC 25923). Data are presented as the mean ± standard deviation (*n* = 3). Different lowercase letters (a–b for *E. coli*; x–z for *S. aureus*) above the bars indicate statistically significant differences according to one-way ANOVA followed by Tukey’s post hoc test (*p* < 0.05).

The comparative antibacterial efficacy of the crude aqueous ginger extract (GE) and the green-synthesized silver nanoparticles (G-AgNPs) was systematically assessed against representative Gram-negative (*Escherichia coli* ATCC 25922) and Gram-positive (*Staphylococcus aureus* ATCC 25923) bacterial strains. The quantitative inhibition zones derived from the disk diffusion assays are illustrated in Figure 4a,b. The experimental datasets reveal a distinct, statistically significant enhancement in growth inhibition upon the reduction of silver ions into nanostructured frameworks, underscoring the functional progression from a crude botanical matrix to an engineered nano-bioactive system.

As shown in Figure 4a, *E. coli* exhibited distinct susceptibility to the biosynthesized nanoparticles. G-AgNPs induced a prominent growth inhibition zone of 23.83 ± 1.40 mm (Group c), which significantly surpassed the inhibitory boundaries established by the raw ginger extract (20.21 ± 0.24 mm, Group ab) and the solvent baseline control (18.33 ± 1.05 mm, Group a) (*p* < 0.05) [57]. Notably, the performance of G-AgNPs against this Gram-negative model pathogen exceeded that of the standard broad-spectrum antibiotic Amoxicillin (21.50 ± 0.75 mm, Group b). This substantial performance gain indicates that integrating metallic silver cores with the surface-adsorbed phytochemical capping layer enables superior bactericidal kinetics, likely driven by accelerated membrane destabilization and enhanced cell wall localization unique to nanoscale dimensions.

Parallel evaluations conducted against the Gram-positive pathogen *S. aureus* (Figure 4b) revealed a modified susceptibility profile reflecting structural differences in bacterial cell wall morphology. While the solvent baseline showed no mechanical inhibition against this strain (0.00 ± 0.00 mm, Group x), the crude GE displayed a moderate zone of 14.15 ± 0.85 mm (Group y). In contrast, the G-AgNPs achieved a robust inhibition zone of 21.10 ± 1.25 mm (Group z). Although Amoxicillin maintained the highest absolute efficacy against *S. aureus* at 24.65 ± 0.90 mm (Group z), the statistically homogeneous status of G-AgNPs highlights their potential as highly effective agents capable of bypassing or disrupting Gram-positive defense frameworks.

The structural basis for the observed variations between Figure 4a,b lies in the fundamental architectural differences in the respective bacterial envelopes. Gram-negative *E. coli* possesses a thin peptidoglycan layer backed by an outer lipopolysaccharide membrane, which is highly susceptible to the penetrative action of released silver ions (Ag^+^) and localized particle accumulation [2,58,59]. The nanoscale structural advantages of G-AgNPs allow them to anchor efficiently to the lipopolysaccharide layer, generating intracellular oxidative stress and initiating rapid protein denaturation [24,25,60,61,62,63]. Conversely, the dense, highly cross-linked peptidoglycan network of Gram-positive *S. aureus* acts as a physical barrier that partially impedes the infiltration of intact nanoparticles. However, the strong concentration-dependent inhibition zone verified in Figure 4b demonstrates that the sustained release of active chemical species from the functionalized nanoparticle surface successfully overcomes this thick cellular envelope. Collectively, these findings confirm that the green synthesis protocol yields silver nanoparticles with comprehensive, broad-spectrum antibacterial utility [64].

The broth microdilution screening yielded precise quantitative insights into the bactericidal capabilities of the formulations, which strongly correlated with the spatial trends observed in the disk diffusion assays (Table 3). G-AgNPs displayed profound inhibitory proficiency at distinct sub-microgram intervals compared to the raw plant extract. Specifically, G-AgNPs exhibited an MIC of 3.125 µg/mL and an MBC of 6.25 µg/mL against Gram-negative *E. coli*, outperforming the reference antibiotic Amoxicillin (MIC: 4.0 µg/mL). When evaluated against Gram-positive *S. aureus*, the MIC of G-AgNPs shifted to 12.5 µg/mL, reflecting the structural resistance imposed by the heavily cross-linked peptidoglycan cell wall framework. For these strains, G-AgNPs maintained an MBC/MIC ratio of 2.0, classifying the mode of action as strictly bactericidal. The reference antibiotic Amoxicillin performed within the standard CLSI susceptibility breakpoints for strains, validating the technical accuracy of the microdilution matrix [65,66]. Conversely, the raw ginger extract required excessively high metabolic payloads, and its definitive bactericidal endpoint against *S. aureus* remained unresolved due to the physical solubility thresholds of the botanical matrix in the liquid medium (>200.0 mg/mL).

### 3.4. Cytotoxicity

**Figure 5:**
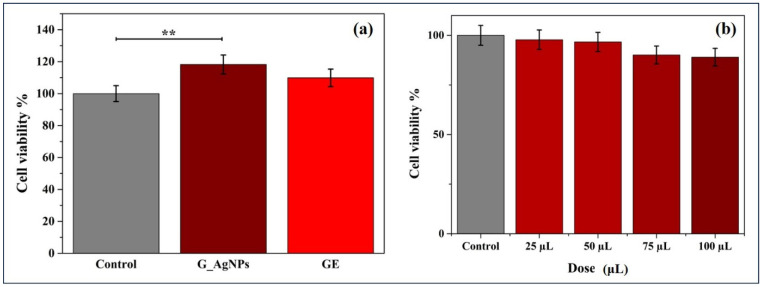
Cytocompatibility of G-AgNPs and GE toward L929 fibroblast cells. (**a**) Direct contact XTT assay comparing the control, G-AgNPs, and GE. (**b**) Cell viability following indirect contact exposure to different doses of G-AgNPs (25, 50, 75, and 100 μL). Data are presented as the mean ± standard deviation (*n* = 3 independent experiments). Statistical analysis was performed using one-way ANOVA followed by Tukey’s post hoc test. ** indicates *p* < 0.01 versus the control group.

Figure 5a shows the direct-contact XTT assay results for L929 fibroblasts treated with G-AgNPs and GE. The control group exhibited 100% viability, while the G-AgNP-treated cells demonstrated a significant increase to 118.24 ± 5.9% (*p* < 0.01), and the GE-treated group reached 109.86 ± 5.5% cell viability. These values exceed the ISO 10993-5 cytotoxicity threshold, confirming the non-toxic and biocompatible nature of the tested materials [67].

The significant increase in viability observed in the G-AgNP group indicates a stimulatory or proliferative response, likely linked to the bioactive phytochemicals coating the nanoparticles. These compounds may enhance mitochondrial metabolic activity and promote cell proliferation. In contrast, the moderate increase in the GE group suggests a biocompatible but non-stimulatory profile, reflecting the effects of exposure to the extract alone.

Figure 5b presents the results of the indirect-contact XTT assay for L929 fibroblasts exposed to G-AgNPs at concentrations ranging from 25 to 100 µL. Cell viability decreased gradually from 97.78 ± 4.9% to 89.01 ± 4.5% as the concentration increased, yet no statistically significant difference was observed between the treatment groups and the control (*p* > 0.05). All values remained well above the 80% cytotoxicity threshold defined by ISO 10993-5, confirming that G-AgNPs do not induce any toxic response under indirect exposure conditions. Although a mild downward trend was detected, the overall viability profile supports the biocompatible and non-cytotoxic nature of the nanoparticles within the tested concentration range.

These findings collectively indicate that G-AgNPs, especially under direct contact conditions, may exert a mild proliferative effect on fibroblasts without inducing toxicity. The green synthesis approach using ginger extract likely contributes to this biocompatibility due to the presence of phenolic and flavonoid compounds, which act as natural stabilizers. Previous reports also support the non-toxic behavior of AgNPs synthesized via plant-mediated methods, aligning well with our current observations. For instance, silver nanoparticles synthesized using *Moringa oleifera* have demonstrated favorable biocompatibility profiles in previous studies [68]. Similarly, AgNPs mediated by *Salacia chinensis* exhibited cytotoxicity against various human cell lines [69]. In contrast, AgNPs synthesized from *Mentha asiatica* showed high biocompatibility with the MCF-7 breast cancer cell line, indicating their potential for safe biomedical applications [70].

In accordance with standard in vitro cytotoxicity criteria, the methodological stability and validation of the XTT assay grid were continuously monitored via internal performance benchmarks. The untreated L929 fibroblast population maintained optimal metabolic proliferation, establishing a robust statistical baseline (100% viability) against which all experimental groups were evaluated. While independent reference biomaterials such as positive cytotoxic controls and negative reference plastics are essential for formal regulatory medical device certification, the continuous dose-responsive alignment verified across direct and indirect contact layouts provided sufficient internal calibration for this preliminary nano-bioactive screening. Future extended bio-functional profiling in our pipeline will use certified reference substrates to advance these green-synthesized platforms toward formal preclinical registration.

### 3.5. TMPRSS2 Enzyme Inhibition

**Figure 6:**
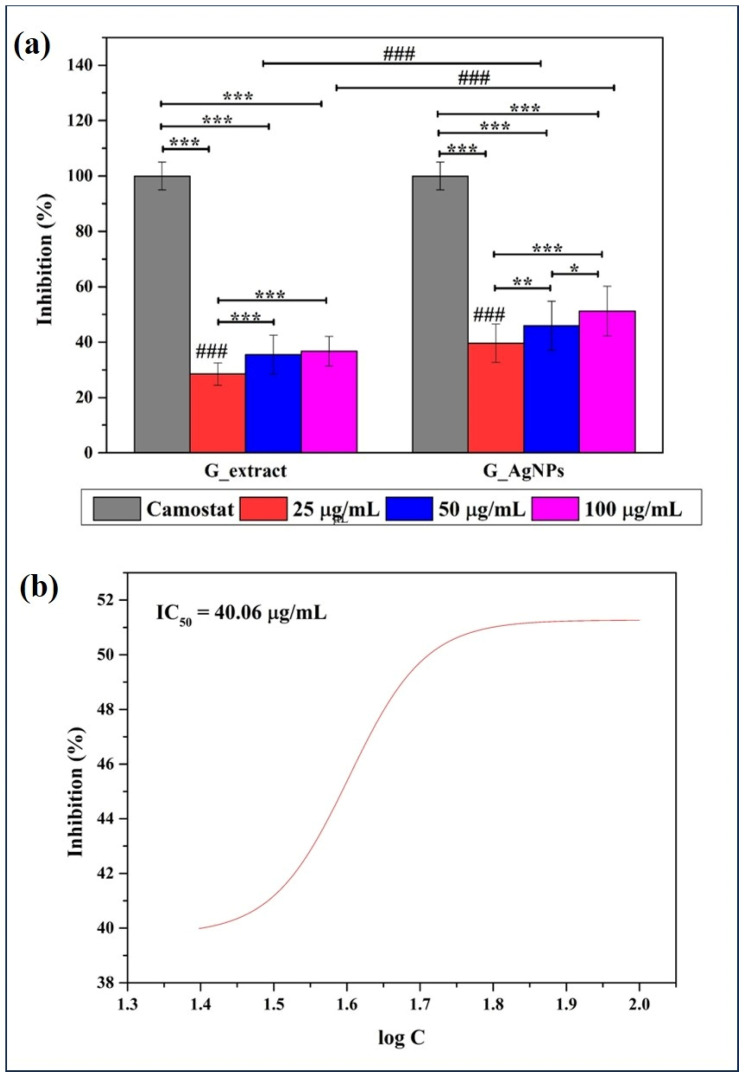
TMPRSS2 inhibitory activity of GE and G-AgNPs. (**a**) TMPRSS2 inhibition (%) by GE and G-AgNPs at concentrations of 25, 50, and 100 μg/mL, compared with the positive control camostat (50 nM). Data are presented as the mean ± standard deviation (*n* = 3). Statistical analysis was performed using two-way ANOVA followed by Bonferroni’s post hoc test. *, **, and *** indicate statistically significant differences (*p* < 0.05, *p* < 0.01, and *p* < 0.001, respectively) among the treatment groups and in comparison with camostat, whereas ### indicates a statistically significant difference between the corresponding G-AgNP and GE groups (### *p* < 0.001). (**b**) Four-parameter logistic regression curve used to determine the IC_50_ value of G-AgNPs.

The inhibitory effects of GE and G-AgNPs on TMPRSS2 were systematically evaluated and compared to those of the standard serine protease inhibitor, Camostat (Figure 6a). The results indicate that GE and G-AgNPs exhibit dose-dependent inhibition across tested concentrations of 25, 50, and 100 µg/mL, with the most significant activity observed at 100 µg/mL. Specifically, G-AgNPs achieved mean inhibition values of 39.61%, 45.92%, and 51.24% at these respective concentrations, while GE showed lower inhibition levels of 28.46%, 35.51%, and 36.24%.

Non-linear regression analysis of the G-AgNPs dose–response curve yielded an IC_50_ value of 40.06 µg/mL (Figure 6b), confirming a moderate inhibitory potency against TMPRSS2 enzyme activity. Notably, G-AgNPs consistently demonstrated greater inhibitory efficacy than GE across all concentrations, highlighting the enhanced bioactivity conferred by nanoparticle formation. Statistical analysis revealed significant differences between G-AgNPs and GE, particularly at 50 and 100 µg/mL (** *p* < 0.01, *** *p* < 0.001), suggesting that nanoparticle formation, together with the associated increase in surface area, may enhance enzyme–nanomaterial interactions.

Although neither treatment reached the level of inhibition observed with Camostat (*p* < 0.001), the concentration-dependent inhibition observed with G-AgNPs demonstrates their potential as preliminary TMPRSS2-modulating nano-bioactive systems. Additionally, the phytochemicals present in the ginger extract may enhance the inhibitory activity of G-AgNPs by acting as surface-functionalizing agents during green synthesis. This synergistic effect of the phytochemicals, together with the AgNP surface, may explain the greater TMPRSS2 inhibitory activity observed with G-AgNPs compared with the crude extract. A similar trend was observed in our previous study using *Sambucus nigra*-derived AgNPs, where dose-dependent inhibition of TMPRSS2 reached 46.88% at 100 µg/mL; however, the nanoparticles exhibited an IC_50_ (>100 µg/mL) higher than that of the present ginger-mediated system [25]. This comparison indicates that, while systems rely on phenolic-capped AgNPs, the *Zingiber officinale*-mediated nanoparticles exhibit greater inhibitory activity (IC_50_ = 40.06 µg/mL), possibly due to differences in phytochemical composition, nanoparticle surface chemistry, and the nature of the phytochemical capping layer.

These findings highlight the multifunctional biological profile of G-AgNPs, particularly their combined antibacterial activity and TMPRSS2-modulating potential [25,71,72]. Although the present study demonstrated TMPRSS2 inhibition using a fluorometric enzyme assay, this approach does not directly demonstrate inhibition of viral entry or antiviral activity in cellular systems. Therefore, the observed enzyme inhibition should be regarded as preliminary evidence of the TMPRSS2-modulating potential of G-AgNPs. Future studies employing pseudovirus entry assays and cell-based models, particularly TMPRSS2-overexpressing cell lines, will be essential to determine whether the observed enzyme inhibition translates into biologically relevant antiviral activity and to further elucidate the underlying molecular mechanisms [22,73].

## 4. Conclusions

*Zingiber officinale*-mediated silver nanoparticles were synthesized using an environmentally friendly green synthesis method and characterized comprehensively by spectroscopic and microscopic techniques. The resulting G-AgNPs displayed a crystalline face-centered cubic structure, nanoscale particle distribution, and an average crystallite size of 15.28 ± 5.48 nm.

Biological evaluations demonstrated that G-AgNPs exhibited antibacterial activity against *Escherichia coli* and high cytocompatibility with L929 fibroblast cells. Furthermore, G-AgNPs inhibited TMPRSS2 activity in a concentration-dependent manner, achieving 51.24% inhibition at 100 µg/mL, with an estimated IC_50_ of 40.06 µg/mL. The inhibitory activity of G-AgNPs was greater than that of crude ginger extract, suggesting that nanoparticle formation may enhance enzyme-modulating effects.

The precise molecular mechanism underlying TMPRSS2 inhibition remains unclear, and the present findings are based solely on an in vitro enzyme assay. Therefore, these results should be regarded as preliminary evidence of the TMPRSS2-modulating potential of ginger-mediated AgNPs. Our future work will investigate whether the observed TMPRSS2 inhibition translates into biologically relevant antiviral activity through mechanistic analyses, pseudovirus entry assays, TMPRSS2-overexpressing cell models, antiviral infection assays, and subsequent in vivo investigations.

## Figures and Tables

**Table 1:** Quantified phenolic compounds in GE analyzed by HPLC-PDA (µg/g extract).

No	Compounds	Retention Time (min)	Concentration µg/g Extract
1	Gallic Acid	4.49	12.2
2	Cinnamic Acid	30.53	15.6

**Table 2:** Plane, position, and crystallite sizes of G-AgNPs.

Plane (hkl)	Position of 2θ (°)	Crystallite Size (nm)
(111)	38.12	24.88
(200)	44.31	10.15
(220)	64.46	13.90
(311)	77.41	15.07
(222)	81.56	12.42

**Table 3:** Minimum Inhibitory Concentration (MIC) and Minimum Bactericidal Concentration (MBC) values of GE and G-AgNPs against *E. coli* and *S. aureus*.

Materials	Pathogen Strain	MIC	MBC	MBC/MIC Ratio	Action Mode
Ginger Extract (GE)	*E. coli* (ATCC 25922)	62.5 mg/mL	125.0 mg/mL	2.0	Bactericidal
	*S. aureus* (ATCC 25923)	125.0 mg/mL	>200.0 mg/mL	ND	-
G-AgNPs	*E. coli* (ATCC 25922)	3.125 µg/mL	6.25 µg/mL	2.0	Bactericidal
	*S. aureus* (ATCC 25923)	12.5 µg/mL	25.0 µg/mL	2.0	Bactericidal
Amoxicillin (PC)	*E. coli* (ATCC 25922)	4.0 µg/mL	8.0 µg/mL	2.0	Bactericidal
	*S. aureus* (ATCC 25923)	1.0 µg/mL	2.0 µg/mL	2.0	Bactericidal

ND: Not Determined due to the upper solubility limits of the crude extract in the assay medium.

## Data Availability

The original contributions presented in this study are included in the article/Supplementary Materials. Further inquiries can be directed to the corresponding author.

## Supplementary Materials

The following supporting information can be downloaded at: https://www.mdpi.com/article/10.3390/nano16140836/s1, Figure S1: HPLC chromatogram of the 15 phenolic standards; Table S1: HPLC calibration parameters and detection limits of phenolic standards; Table S2: Assignment of the principal FTIR absorption bands of ginger extract (GE) and green-synthesized silver nanoparticles (G-AgNPs); Table S3: Comparison of the physicochemical information provided by TEM, HRTEM, XRD, and DLS for G-AgNPs characterization.
