# Supplementary material for: Green-Synthesized Silver Nanoparticles from Zingiber officinale: Physicochemical Characterization, Antibacterial Activity, and TMPRSS2-Modulating Potential
Source: Nanomaterials (Basel). 2026 Jul 8;16(14):836. doi: 10.3390/nano16140836 (PMC13415599; doi:10.3390/nano16140836)
Supplement: Supplementary file 1 [file nanomaterials-16-00836-s001.zip › nanomaterials-4401585-supplementary.pdf]

Supplementary Materials

Green-Synthesized Silver Nanoparticles from *Zingiber officinale*: Physicochemical Characterization, Antibacterial Activity, and TMPRSS2-Modulating Potential

Ozlem Tavukcuoglu [1], Fatih Ciftci [2,3,4], Nilüfer Evcimen Duygulu [5], Duygu Misirli [1], Mahfuz Elmastaş [1] and Ahmet Akif Kızılkurtlu [6,*]

[1] Department of Biochemistry, Faculty of Hamidiye Pharmacy, University of Health Sciences, Istanbul 34668, Turkey; ozlemoztolan@gmail.com (O.T.); duygu.misirli@sbu.edu.tr (D.M.); mahfuz.elmastas@sbu.edu.tr (M.E.)
[2] Faculty of Engineering, Department of Biomedical Engineering, Fatih Sultan Mehmet Vakıf University, Istanbul 34445, Turkey; fciftci@fsm.edu.tr
[3] Biomedical Electronic Design Application and Research Center (BETAM), Fatih Sultan Mehmet Vakıf University, Istanbul 34445, Turkey
[4] BioriginAI Research Group, Department of Biomedical Engineering, Fatih Sultan Mehmet Vakıf University, Istanbul 34445, Turkey
[5] Faculty of Chemical and Metallurgical Engineering, Department of Metallurgical and Material Engineering, Yildiz Technical University, Istanbul 34469, Turkey; nevci@yildiz.edu.tr
[6] Faculty of Engineering and Natural Sciences, Department of Biomedical Engineering, Atlas University, Istanbul 34408, Turkey
* Correspondence: ahmet.kizilkurtlu@atlas.edu.tr

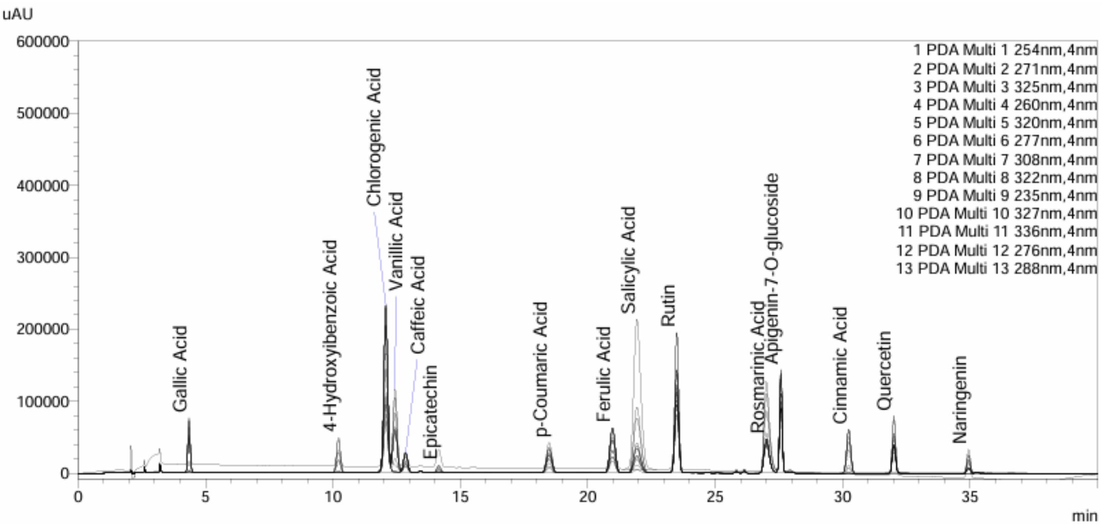

Figure S1. HPLC chromatogram of the 15 phenolic standards

Table S1. HPLC calibration parameters and detection limits of phenolic standards

No	Name	Retention Time	Unit	Maximum Wavelength	Standard calibration equation	R^2	Limit of Detection (LOD)	Quantification of Detection (QOD)
1	Gallic acid	4.352	mg/L	271nm	y=29799.9x+6494.60	0.9995969	0.744081	2.254791
2	4-hydroxybenzoic acid	10.217	mg/L	254nm	y=40036.7x+1238.33	0.9995870	0.644531	1.953125
3	Chlorogenic acid	12.073	mg/L	325nm	y=28066.0x+25870.2	0.9990219	1.368727	4.147657
4	Vanillic acid	12.437	mg/L	260nm	y=48654.4x-26981.1	0.9997661	1.056564	3.201708
5	Caffeic acid	12.850	mg/L	320nm	y=16914.9x-1409.46	0.9972149	3.173077	9.615385
6	Epicatechin	14.150	mg/L	277nm	y=4788.08x+789.457	0.9986685	8.461538	25.64103
7	p-coumaric acid	18.486	mg/L	308nm	y=64013.4x-19190.2	0.9933422	1.380753	4.1841
8	Ferulic acid	20.971	mg/L	322nm	y=46665.0x-14606.2	0.9928040	1.386555	4.201681
9	Salicylic acid	21.929	mg/L	235nm	y=23472.2x+25113.1	0.9994182	4.690635	14.21405
10	Rutin	23.494	mg/L	254nm	y=17392.1x-5957.13	0.9959336	2.125867	6.442022
11	Rosmarinic acid	26.857	mg/L	327nm	y=34304.9x-12772.0	0.9999601	1.005331	3.046458
12	Apigenin-7-O-glucoside	27.574	mg/L	336nm	y=39321.9x+1685.18	0.9993868	0.72955	2.210759

13	Cinnamic acid	30.234	mg/L	276nm	y=75026.0x-11276.0	0.9988811	0.677618	2.053388
14	Quercetin	32.008	mg/L	254nm	y=26403.4x+1558.71	0.9997869	1.272494	3.856041
15	Naringenin	34.939	mg/L	288nm	y=24207.0x+2212.93	0.9920277	1.314741	3.984064

Table S2. Assignment of the principal FTIR absorption bands of ginger extract (GE) and green-synthesized silver nanoparticles (G-AgNPs)

GE (cm^{-1})	G-AgNPs (cm^{-1})	Band assignment	Interpretation
3326	3324	O–H stretching (alcohols and phenolic compounds)	Slight shift indicates involvement of hydroxyl-containing phytochemicals during AgNP formation.
2988	**2972, 2927, 2881**	Aliphatic C–H stretching	The appearance of additional bands suggests the participation of aliphatic phytochemicals in nanoparticle formation.
2131	–	C≡C or C≡N stretching	Band detected only in the GE spectrum.
1635	1637	C=O stretching (amide I/conjugated carbonyls)	Slight shift indicates interaction of carbonyl-containing biomolecules with the nanoparticle surface.
1406	–	C–H bending	Detected in the GE spectrum.
1394	1379	C–N stretching / aromatic vibrations	Shift reflects changes in the local chemical environment after nanoparticle formation.
1250	1327	Phenolic ring vibrations	Changes suggest the participation of phenolic compounds in nanoparticle stabilization.
1066, 1057	1087, 1046	C–O–C / C–OH stretching	Shifts indicate adsorption of oxygen-containing phytochemicals onto the AgNP surface.
–	**1454, 1418**	Carbonyl- and hydroxyl-containing biomolecules	Additional bands observed only in G-AgNPs.
591	630	Aromatic C–H out-of-plane bending / skeletal vibrations	The shift suggests a structural rearrangement following AgNP formation.
–	**879, 803**	Fingerprint region vibrations	Additional fingerprint bands were observed following nanoparticle formation.
–	**432**	Ag–O vibration/metal–ligand interaction (tentative)	Tentatively supports interactions between silver nanoparticles and plant-derived biomolecules.

Table S3. Comparison of the physicochemical information provided by TEM, HRTEM, XRD, and DLS for G-AgNPs characterization

Technique	Parameter measured	Result	Characteristics of the measurement
TEM	Primary particle size	10.61 ± 1.31 nm	Direct imaging of individual nanoparticles in the dry state.
HRTEM	Crystal lattice (lattice fringes)	FCC lattice fringes	Confirms crystallinity at atomic resolution.
XRD	Crystallite size	15.28 nm	Measures coherent crystalline domains rather than the overall particle size.
DLS	Hydrodynamic diameter	80.06 ± 9.80 nm	Measures the hydrodynamic diameter of nanoparticles in suspension, including the metallic core together with the phytochemical coating, hydration shell, and possible mild aggregation.
